# lncRNA TMPO antisense RNA 1 promotes the malignancy of cholangiocarcinoma cells by regulating let-7g-5p/ high-mobility group A1 axis

**DOI:** 10.1080/21655979.2022.2025700

**Published:** 2022-01-18

**Authors:** Hongbin Chang, Yixin Yao

**Affiliations:** a Department of General Surgery, Hanyang Hospital Affiliated to Wuhan University of Science and Technology, Wuhan, China; b Department of General Surgery, Wuhan Hanyang Hospital, Wuhan, China

**Keywords:** Cholangiocarcinoma, TMPO-AS1, let-7g-5p, HMGA1

## Abstract

Cholangiocarcinoma (CHOL) is often diagnosed at an advanced stage; therefore, exploring its key regulatory factors is important for earlier diagnosis and treatment. This study aimed to identify the mechanisms of long non-coding RNA (lncRNA) TMPO Antisense RNA 1 (TMPO-AS1), microRNA let-7 g-5p, and high-mobility group A1 (HMGA1) proteins in CHOL. Our results, through quantitative real-time PCR and Western blot detection, showed that TMPO-AS1 and HMGA1 were overexpressed while let-7 g-5p was underexpressed in CHOL. Cell function experiments in CHOL cells revealed that TMPO-AS1 knockdown inhibited cell proliferation, colony formation, and cell migration, but induced apoptosis. TMPO-AS1 knockdown also suppressed tumor growth in vivo. Together with luciferase assay and Western blotting, we found that TMPO-AS1 could sponge let-7 g-5p to promote HMGA1 expression. Moreover, HMGA1 overexpression attenuated the effect of TMPO-AS1 downregulation in CHOL cells. Overall, our findings identified the oncogenic effect of TMPO-AS1 on CHOL cells, which may put forward a novel methodology for CHOL diagnosis and therapy.

## Graphical abstract

**Figure:**
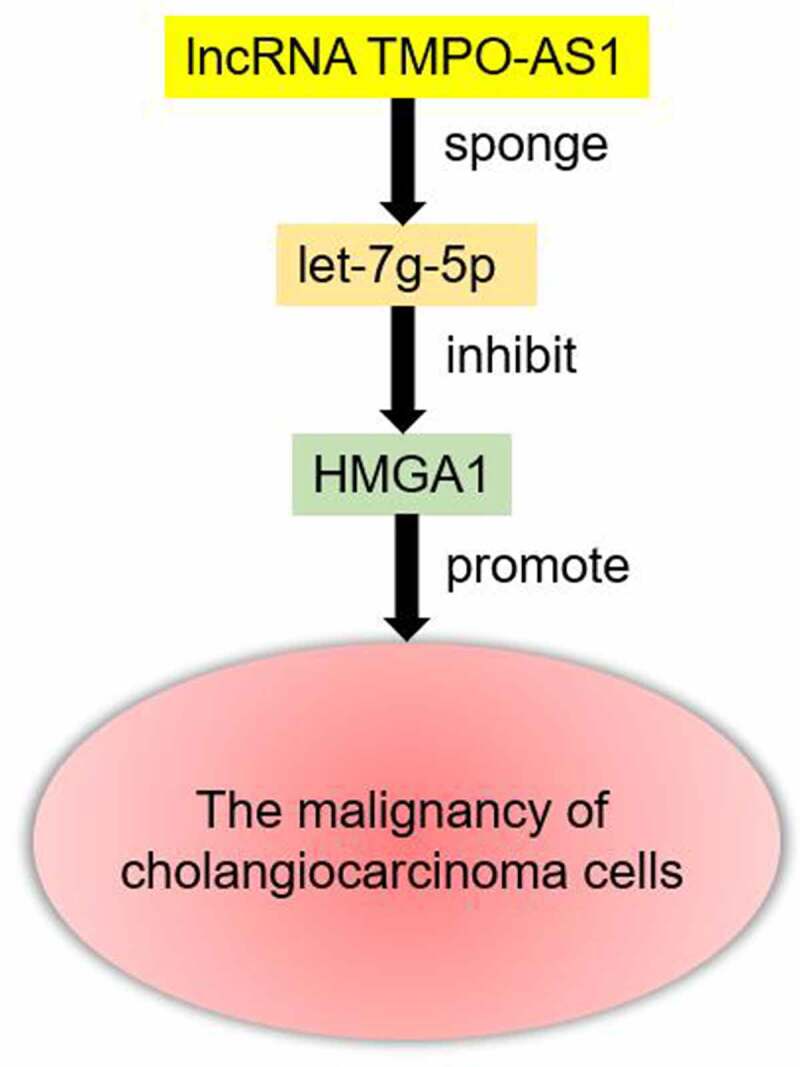

## Introduction

Cholangiocarcinoma (CHOL) originates from the epithelial cells of the bile duct and is a highly invasive malignant tumor [1]. Recently, the incidence and mortality of CHOL have increased worldwide [2]. Although pharmacological interventions for CHOL have improved, radical resection remains to be the most effective treatment for this disease [3]. Moreover, many patients diagnosed with CHOL are at an advanced stage due to its insidious onset [4,5]. Therefore, understanding the underlying mechanisms and identifying key biomarkers of CHOL progression is important for better diagnosis and therapeutic options.

Long non-coding RNAs (lncRNAs) are reported to be regulatory factors that participate in cancer progression by sponging microRNAs (miRNAs) to affect the expression of cancer-related genes; although, lncRNAs cannot encode proteins [6–8]. In CHOL, an increasing number of studies have revealed that some lncRNAs are the oncogenic regulators for CHOL development, such as lncRNA TTN-AS1 [9], lncRNA TUG1 [10] and lncRNA PCAT6 [11]. Furthermore, some lncRNAs are the anti-tumor regulators for CHOL development, such as lncRNA NEF [12], lncRNA MEG3 [13] and lncRNA CASC2 [14]. TMPO Antisense RNA 1 (TMPO-AS1), a member of the lncRNA family, promotes the progression of multiple cancers, including bladder cancer [15], cervical cancer [16] and lung cancer [17]. Nevertheless, the function of TMPO-AS1 in CHOL has not yet been investigated.

miRNAs are a well-known group of small non-coding RNAs that bind to the 3’-untranslated region (3’-UTR) of target genes and regulate cancer progression [18]. For example, miR-106-5p targets CNN1 to regulate the Rho/ROCK1 pathway, thereby contributing to the metastasis of breast cancer [19]. miR-4319 is an anti-tumor miRNA in colorectal cancer that binds to the ABTB1 3’-UTR [20]. let-7 g-5p, another miRNA, has been reported to suppress the malignancy of nasopharyngeal carcinoma [21] and epithelial-mesenchymal transition in glioblastoma [22]. However, the effect and mechanism of let-7 g-5p on CHOL are still unclear.

High-mobility group A (HMGA) proteins, consisting of HMGA1a, HMGA1b, HMGA1c, and HMGA2, are small nuclear proteins with high mobility [23]. HMGA1 was first discovered by Lund et al. [24] and is an oncogene in cervical cancer. Further studies have revealed the oncogenic function of HMGA1 in colorectal cancer [25], gastric cancer [26], and breast cancer [27]. In 2017, Quintavalle C et al. [28] provided evidence that HMGA1 was overexpressed in 51% of CHOL patient samples, and HMGA1 overexpression promoted proliferation, colony formation, and resistance to gemcitabine treatment. Another study in 2021 found that HMGA1 promotes xenograft tumor growth and radioresistance in CHOL [29]. However, the upstream role of HMGA1 in CHOL has not yet been discovered.

In this study, we aimed to explore the function and mechanism of TMPO-AS1 in CHOL. Together with bioinformatics analyses, we hypothesized that the let-7 g-5p/HMGA1 axis was downstream of TMPO-AS1. Our findings may enrich the regulatory network of CHOL progression and provide a novel model for CHOL diagnosis and therapy.

## Material and methods

### Bioinformatics prediction

TMPO-AS1 expression in human cancers was analyzed according to the TCGA database. Then, the upregulated differentially expressed genes (DEGs) in CHOL were screened from the GEPIA database and an mRNA microarray GSE77984 with the screening criteria of adjusted P (adj. P) < 0.05 and logFC > 2. The levels of the screened genes were analyzed according to the TCGA database. The correlation between TMPO-AS1 and the key gene HMGA1 was analyzed using starBase according to the data from TCGA. Finally, the miRNAs binding to TMPO-AS1 were predicted using starBase, whereas TargetScan and miRWalk predicted the miRNAs binding to HMGA1.

### Clinical samples collection and cell culture

CHOL tissues and paired adjacent normal bile duct tissues were obtained from 36 patients who had been diagnosed with CHOL in our hospital between October 2019 and March 2021. Our study was approved by the ethics committee of Hanyang Hospital. Informed consent was obtained from all subjects, and the clinical characteristics of all subjects are listed in Table 1.

**Table 1.** The clinical characteristics of 36 cholangiocarcinoma patients

Characteristics	Value [(n (%)]
Gender	
Male	20 (55.6%)
Female	16 (44.4%)
Age (years)	
≤ 60	19 (52.8%)
>60	17 (47.2%)
Location	
Intrahepatic	13 (36.1%)
Perihilar	14 (38.9%)
Distal	9 (25.0%)
TNM stage	
I–II	22 (61.1%)
III–IV	14 (38.9%)
Neoplasm histologic grade	
G1-G2	26 (72.2%)
G3-G4	10 (27.8%)

All cell lines were purchased from BeNa Culture Collection (China), including CHOL cell lines (HCCC9810, HuCCT1, and RBE) and the normal human biliary epithelial cell line HIBEC. HIBEC, HCCC9810, and HuCCT1 cells were cultured in RPMI-1640 medium, while RBE cells were cultured in DMEM. All cells were cultured in 10% FBS and maintained in a humidified incubator under 5% CO_2_ and 37°C.

### Cell transfection

Two short hairpin RNAs targeting TMPO-AS1 (sh-TMPO-AS1-1 and sh-TMPO-AS1-2), let-7 g-5p mimic, and their negative control (sh-NC and mimic-NC) were provided by RiboBio Co., Ltd. (China). pcDNA3.1-HMGA1 overexpression vectors were also synthesized by RiboBio Co., Ltd. using empty pcDNA3.1 vectors as a negative control. HuCCT1 and RBE cells were transfected with 50 nM sh-TMPO-AS1-1/sh-TMPO-AS1-2, 50 nM let-7 g-5p mimic, 50 nM pcDNA3.1-HMGA1, and their negative controls using Lipofectamine 3000 (Thermo Fisher Scientific, USA).

### Quantitative real-time PCR (qRT-PCR)

Trizol reagents, purchased from Thermo Fisher Scientific (USA), were used to isolate total RNA from tissues and cells. For miRNA, 1 μg of RNA was reverse transcribed with the miScript II RT kit (QIAGEN, Germany) after which it underwent qRT-PCR using the miScript SYBR Green PCR Kit (QIAGEN). For lncRNA and mRNA, 1 μg of RNA was reverse transcribed using the TaKaRa PrimeScript RT reagent (Japan) and qRT-PCR was performed using SYBR Green (Biotool, Switch). The 2^−ΔΔct^ method [30] was used to analyze the relative levels of lncRNAs, miRNAs, and mRNAs with the internal references of either GAPDH or U6. Primer sequences are listed in Table 2.

**Table 2.** The primer sequences used in the study

Gene Name	Sequence (5’ to 3’)
TMPO-AS1	Forward: CCTCCTGCCTGTAGTGTGTG
	Reverse: CCAGACCCGGACACAAAAGA
Let-7 g-5p	Forward: GCACTGAGTTAGTAGGTGGT
	Reverse: GATCCAGTTTTTTTTTTTTTTTAACTATGC
HMGA1	Forward: TCCAGGAAGGAAACCAAGG
	Reverse: AGGACTCCTGCGAGATGC
U6	Forward: CTCGCTTCGGCAGCACA
	Reverse: AACGCTTCACGAATTTGCGT
GAPDH	Forward: GGAGCGAGATCCCTCCAAAAT
	Reverse: GGCTGTTGTCATACTTCTCATGG

### Cell counting kit-8 (CCK8) assay

The CCK8 kit (Beyotime, China) was used to assess the change in cell proliferation, which is often used to assess the proliferation of CHOL cells [31]. Briefly, HuCCT1 and RBE cells were seeded into 96-well plates at a density of 4 × 10^3^ cells per well. After transfection at 0, 24, 48, and 72 h, CCK8 solution was added to the 96-well plate at 10 μL per well. After incubating for two hours, the optical density (OD) was measured at 450 nm with a microplate reader to draw the growth curve.

### Colony formation assay

A total of 400 CHOL cells per well were seeded into 12-well plates and kept in a humidified incubator under 5% CO_2_ and 37°C. Cell transfection was performed at 48 h intervals. After two weeks, visible colonies were fixed and stained with crystal violet. Finally, the colonies were photographed under a light microscope. This assay was performed according to a previous study [32].

### Flow cytometry

The change in apoptosis rate in HuCCT1 and RBE cells was assessed by flow cytometry using the Annexin V-FITC/PI Apoptosis Detection Kit (Yeasen Biotechnology (Shanghai) Co., Ltd., China) as described previously [10]. Briefly, after transfection, 1 × 10^6^ cells were digested with 0.25% trypsin, rinsed with pre-cooled PBS, and added to the binding buffer. Subsequently, Annexin V-FITC and propidium iodide were added to the cells, which were then incubated for 15 min at 22°C in the dark. The apoptosis rate was measured using a FACSCalibur flow cytometer (BD Biosciences, USA).

### Wound healing assay

The transfected CHOL cells (1 × 10^4^) were seeded into 6-well plates and incubated until more than 90% confluence was reached. Then, the monolayer cells were scratched using a 200 μL sterile micropipette tip. After removing the cell debris, the cells were incubated in a serum-free medium for 24 h. Finally, the images of wound closure at 0 and 24 h were photographed using a light microscope at 100× magnification. This assay was performed according to a previous study [33].

### In vivo tumorigenesis assay

Four-week-old female BALB/c nude mice were obtained from Cyagen Biosciences (China) for us to perform an in vivo tumorigenesis assay that was approved by the Institute Animal Ethics Committee. We stably transfected 1 × 10^7^ HuCCT1 cells with either TMPO-AS1 shRNA lentiviral vector (sh-lnc) or negative control (sh-NC), which were both purchased from GenePharma (Shanghai, China). The transfected cells were subcutaneously injected into the flank of randomly assigned nude mice (sh-lnc group and sh-NC group, n = 5/group). Mice were euthanized 28 days after injection, and the tumors were subsequently removed for volume and mass calculations. All experimental protocols were based on a previous study [34].

### Luciferase assay

The binding sites in TMPO-AS1 (5’-CUACCUC-3’) and HMGA1 3’-UTR (3’-ACAAACUACCUC-5’) for let-7 g-5p were predicted using starBase and TargetScan, respectively. According to the predictive results, the wild type TMPO-AS1 or HMGA1 (TMPO-AS1-WT or HMGA1-WT) with the binding sites and the mutant TMPO-AS1 or HMGA1 (TMPO-AS1-MUT or HMGA1-MUT) without the binding sites were constructed into the pGL3 vector. Then, TMPO-AS1-WT/HMGA1-WT and TMPO-AS1-MUT/HMGA1-MUT were transfected into HuCCT1 and RBE cells together with let-7 g-5p mimic or negative control (mimic-NC). After cell transfection, a luciferase reporter gene detection kit (Promega, USA) was used to measure luciferase activity according to the supplier’s standards.

### Western blotting

According to a previous study description [9], CHOL cells were lysed with RIPA buffer (Beyotime, China) to isolate total protein content, and the concentration of isolated protein was measured using a BCA protein kit (Pierce, USA). Then, 20 μg of total protein was separated by 12% SDS-PAGE, followed by transfer onto PVDF membranes. After blocking the membranes with 5% nonfat milk, the membranes were incubated with primary antibodies including the HMGA1 antibody (ab129153) and the GAPDH antibody (ab9485) at 4°C overnight. After incubation with the primary antibodies, the membranes were incubated with a rabbit IgG antibody (ab270144). The protein was visualized using an enhanced chemiluminescence detection kit (Millipore, USA) and exposed to X-ray film. All antibodies were purchased from Abcam (Cambridge, UK).

### Statistical analysis

All data are shown as the mean value ± SD from three independent experiments. The differences in the expression of TMPO-AS1, HMGA1, and let-7 g-5p in CHOL and paired normal tissues were analyzed by a paired Student’s t-test. Other differences between more than two groups were analyzed using one-way or two-way ANOVA. Statistical significance was set at p < 0.05.

## Results

In this study, we aimed to explore the effects and mechanisms of TMPO-AS1 in CHOL. Together with bioinformatics analysis and cell functional experiments, it was found that TMPO-AS1 was an oncogenic lncRNA in CHOL by sponging the let-7 g-5p/HMGA1 axis. Our study enriched the regulatory mechanism of lncRNAs in CHOL.

### TMPO-AS1 was a key lncRNA in CHOL

**Figure 1.**
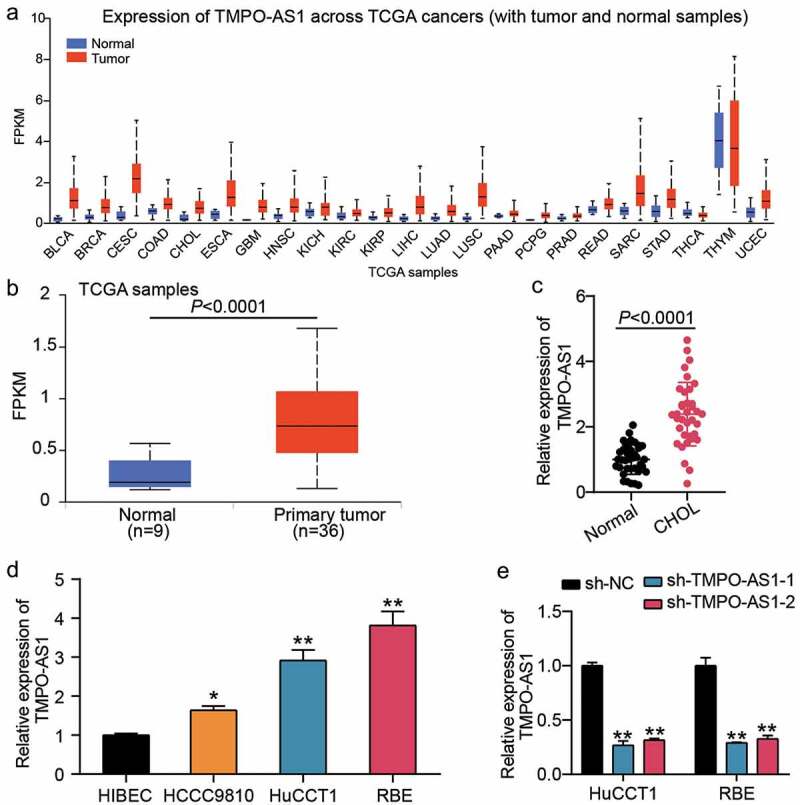
TMPO-AS1 was a key lncRNA in CHOL. (a) TCGA showed expression of TMPO-AS1 in multiple human cancers. (b) TCGA showed the expression of TMPO-AS1 in CHOL. (c) qRT-PCR detected the expression of TMPO-AS1 in CHOL tissues and normal tissues. (d) qRT-PCR detected the expression of TMPO-AS1 in CHOL cell lines (HCCC9810, HuCCT1 and RBE) and human intrahepatic biliary epithelial cell line (HIBEC). *P < 0.05, **P < 0.01 vs. HIBEC. (e) qRT-PCR identified the transfection efficiency of sh-TMPO-AS1-1 and sh-TMPO-AS1-2. sh-TMPO-AS1-1 and sh-TMPO-AS1-2 were two siRNAs targeting TMPO-AS1. NC, negative control. **P < 0.01 vs. sh-NC.

Based on the data from TCGA, it was found that TMPO-AS1 was upregulated in almost all human cancer types (Figure 1(a)), and its expression was elevated by 3-fold in CHOL samples compared with normal non-tumor samples (Figure 1(b)). In tissue samples collected from 36 patients with CHOL, TMPO-AS1 expression increased by approximately 3-fold in CHOL samples compared with adjacent normal samples (Figure 1(c)). In cells, TMPO-AS1 was found to be overexpressed in CHOL cells, especially in HuCCT1 and RBE cells (Figure 1(d)). Hence, HuCCT1 and RBE cells were selected for transfection with sh-TMPO-AS1-1 or sh-TMPO-AS1-2. The results showed that sh-TMPO-AS1-1 and sh-TMPO-AS1-2 successfully downregulated TMPO-AS1 expression by more than 60% (Figure 1(e)). Our data shows that TMPO-AS1 is overexpressed in CHOL tissues and cells.

### TMPO-AS1 knockdown negatively regulated the malignancy of CHOL

**Figure 2.**
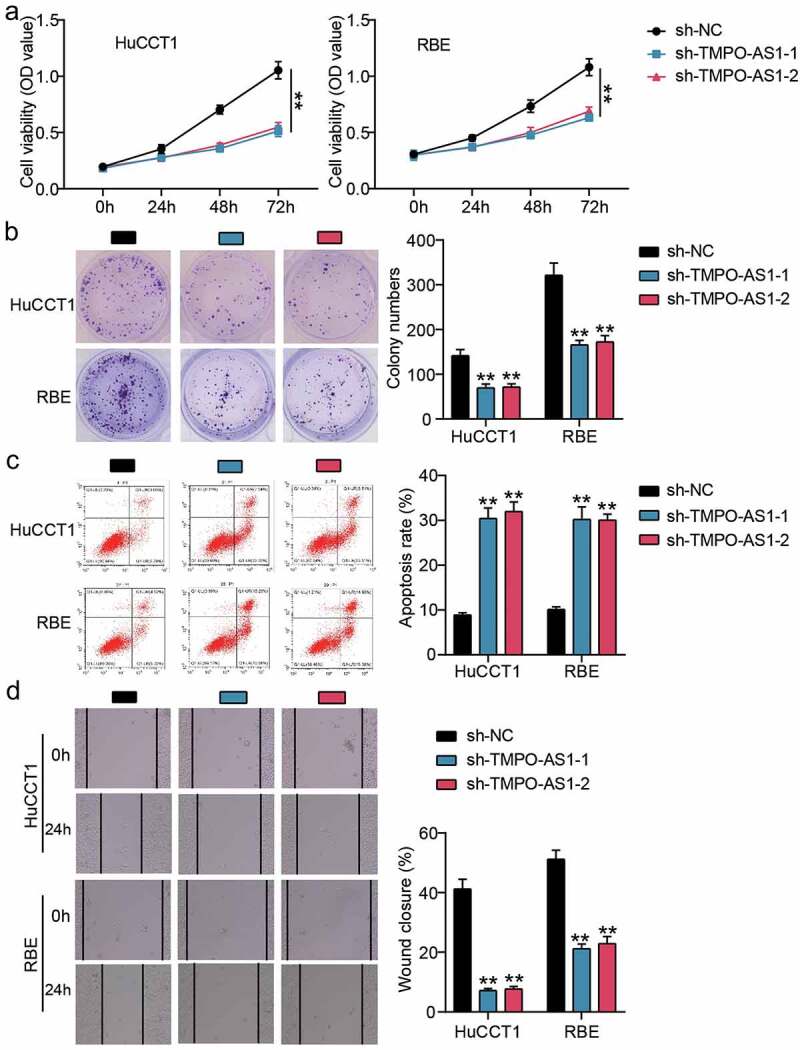
TMPO-AS1 knockdown negatively regulated the malignancy of CHOL cells. (a) CCK8 assay detected cell proliferation in HuCCT1 and RBE cells. (b) Colony formation assay assessed the colony formation capability of HuCCT1 and RBE cells. (c) Flow cytometry assay measured the cell apoptosis rate of HuCCT1 and RBE cells. (d) Wound healing assay identified the change of cell migration in HuCCT1 and RBE cells. sh-TMPO-AS1-1 and sh-TMPO-AS1-2 were two siRNAs targeting TMPO-AS1. NC, negative control. **P < 0.01 vs. sh-NC.

**Figure 3.**
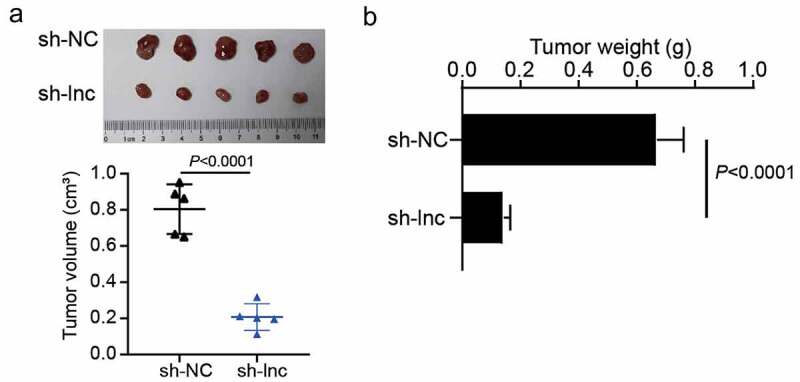
The effect of TMPO-AS1 on CHOL cells tumorigenesis in vivo. (a) The effect of silencing TMPO-AS1 on tumor volume in nude mice. (b) The effect of silencing TMPO-AS1 on tumor weight in nude mice. sh-lnc, TMPO-AS1 shRNA. sh-NC, negative control.

We performed a series of cell function assays to investigate the effects of TMPO-AS1 on CHOL cells. The CCK8 assay revealed that TMPO-AS1 knockdown impaired the proliferation of CHOL cells (Figure 2(a)). After performing the colony formation assay, it was found that the colony formation capability was reduced by half in CHOL cells with TMPO-AS1 knockdown (Figure 2(b)). For cell apoptosis, TMPO-AS1 knockdown increased the apoptosis rate by 3-fold in both HuCCT1 and RBE cells (Figure 2(c)). Cell migration, assessed by a wound healing assay, was impaired when CHOL cells were transfected with sh-TMPO-AS1-1 or sh-TMPO-AS-2 (Figure 2(d)). In vivo, silencing TMPO-AS1 reduced tumor volume (Figure 3(a)) and tumor weight (Figure 3(b)), suggesting that silencing TMPO-AS1 inhibits tumor growth in vivo. Taken together, these data illuminate the effect of reduced CHOL malignancy through TMPO-AS1 knockdown.

### Let-7 g-5p/HMGA1 axis was downstream of TMPO-AS1

**Figure 4.**
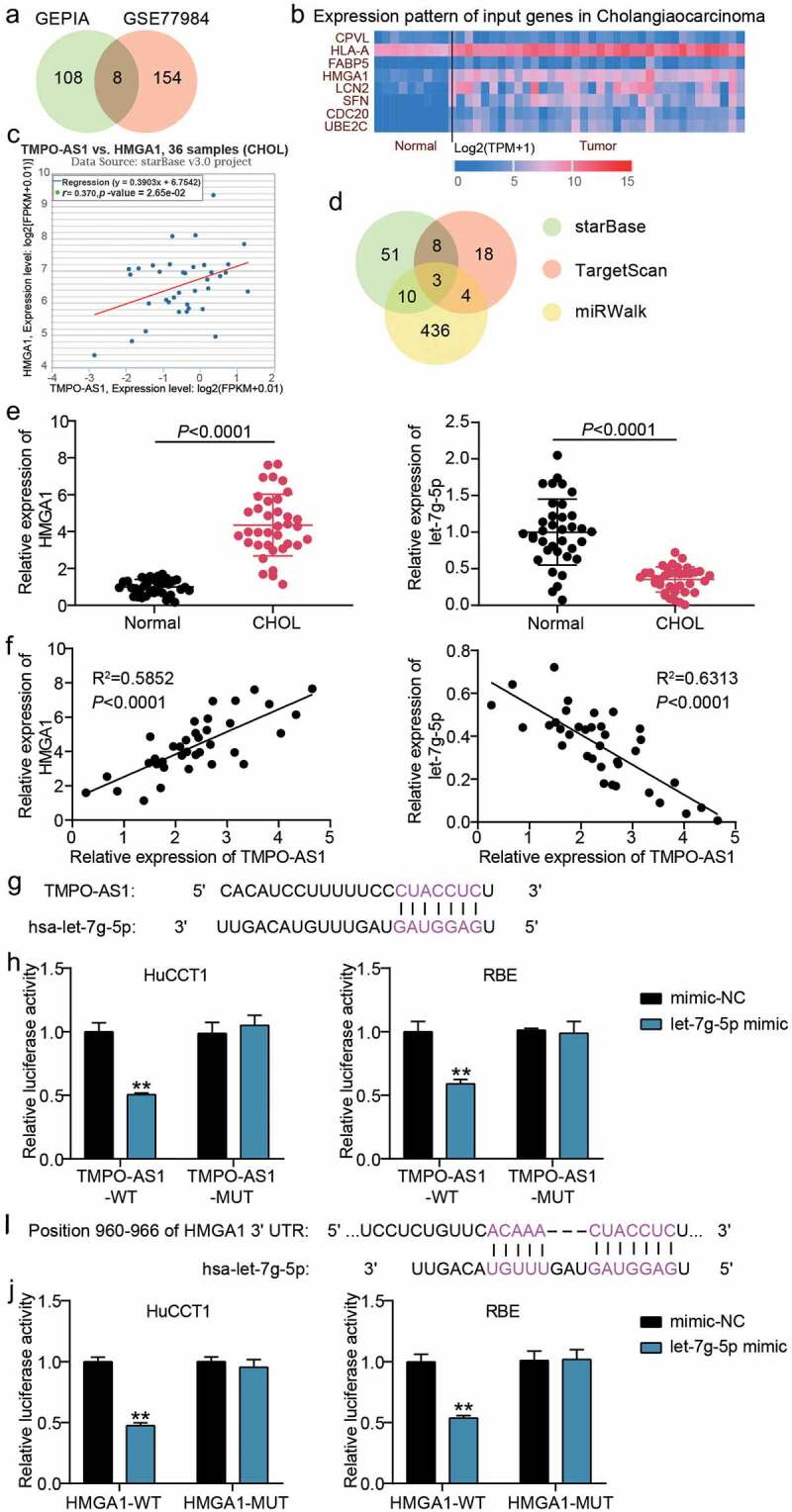
Let-7 g-5p/HMGA1 axis was the downstream of TMPO-AS1. (a) 8 genes were overlapped from GEPIA and GSE77984 with the screening criteria of adj.P < 0.05 and logFC>2. (b) The expression of 8 selected genes in normal and CHOL samples according to TCGA data. (c) HMGA1 expression was positively correlated to TMPO-AS1 expression in CHOL samples according to TCGA data. (d) Three miRNAs including let-7 g-5p, let-7a-5p and let-7d-5p were the common miRNAs in starBase, TargetScan and miRWalk. (e) qRT-PCR detected the expression of HMGA1 and let-7 g-5p in CHOL tissues and normal tissues. (f) Pearson’s correlation analysis revealed the correlation between TMPO-AS1, HMGA1 and let-7 g-5p in CHOL samples. (g) The binding sites between TMPO-AS1 and let-7 g-5p were predicted by starBase. (h) The luciferase assay proved the targeting relationship between let-7 g-5p and TMPO-AS1 in CHOL samples. WT, wild-type. MUT, mutant. **P < 0.01 vs. co-transfection of TMPO-AS1-WT and mimic-NC. (i) TargetScan showed the binding sited between HMGA1 3ʹUTR and let-7 g-5p. (j) The luciferase assay proved the targeting relationship between let-7 g-5p and HMGA1 3ʹUTR. WT, wild-type. MUT, mutant. **P < 0.01 vs. co-transfection of HMGA1-WT and mimic-NC.

GEPIA and GSE77984 stored DEGs in CHOL samples, so they were used to screen the key genes. Using adj. P < 0.05 and logFC > 2 as the screening criteria, eight genes were overlapped from GEPIA and GSE77984 (Figure 4(a)). According to TCGA data, HMGA1 was found to be upregulated in CHOL samples (Figure 4(b)), while HMGA1 levels were positively correlated with TMPO-AS1 levels in the samples, too (Figure 4(c)). Consequently, we selected HMGA1 as the gene of interest. Thereafter, miRNAs binding to TMPO-AS1 were predicted by starBase, while miRNAs binding to HMGA1 3’-UTR were predicted by TargetScan and miRWalk. The results showed that let-7 g-5p, let-7a-5p, and let-7d-5p overlapped with starBase, miRWalk, and TargetScan predictions (Figure 4(d)). Let-7 g-5p was selected as our miRNA of interest, owing to its limited study in CHOL. After performing qRT-PCR, HMGA1 was found overexpressed in the collected CHOL samples, whereas let-7 g-5p expression was downregulated (Figure 4(e)). Pearson’s correlation analysis revealed that HMGA1 and let-7 g-5p expression were positively and negatively correlated with TMPO-AS1 expression in the CHOL samples (Figure 4(f)). The binding sites between TMPO-AS1 and let-7 g-5p are shown in Figure 4(g). The luciferase assay verified that let-7 g-5p could bind to TMPO-AS1 due to the decrease in luciferase activity following co-transfection of TMPO-AS1-WT and let-7 g-5p mimic groups (Figure 4(h)). Moreover, TargetScan revealed the binding site between HMGA1 3’-UTR and let-7 g-5p (Figure 4(i)). The luciferase assay also proved that let-7 g-5p could bind to HMGA1 3’-UTR due to the decrease in luciferase activity in co-transfection of HMGA1-WT and let-7 g-5p mimic groups (Figure 4(j)). These results indicate that TMPO-AS1 could sponge let-7 g-5p to regulate HMGA1 expression.

### HMGA1 overexpression relieved the effect of sh-TMPO-AS1

**Figure 5.**
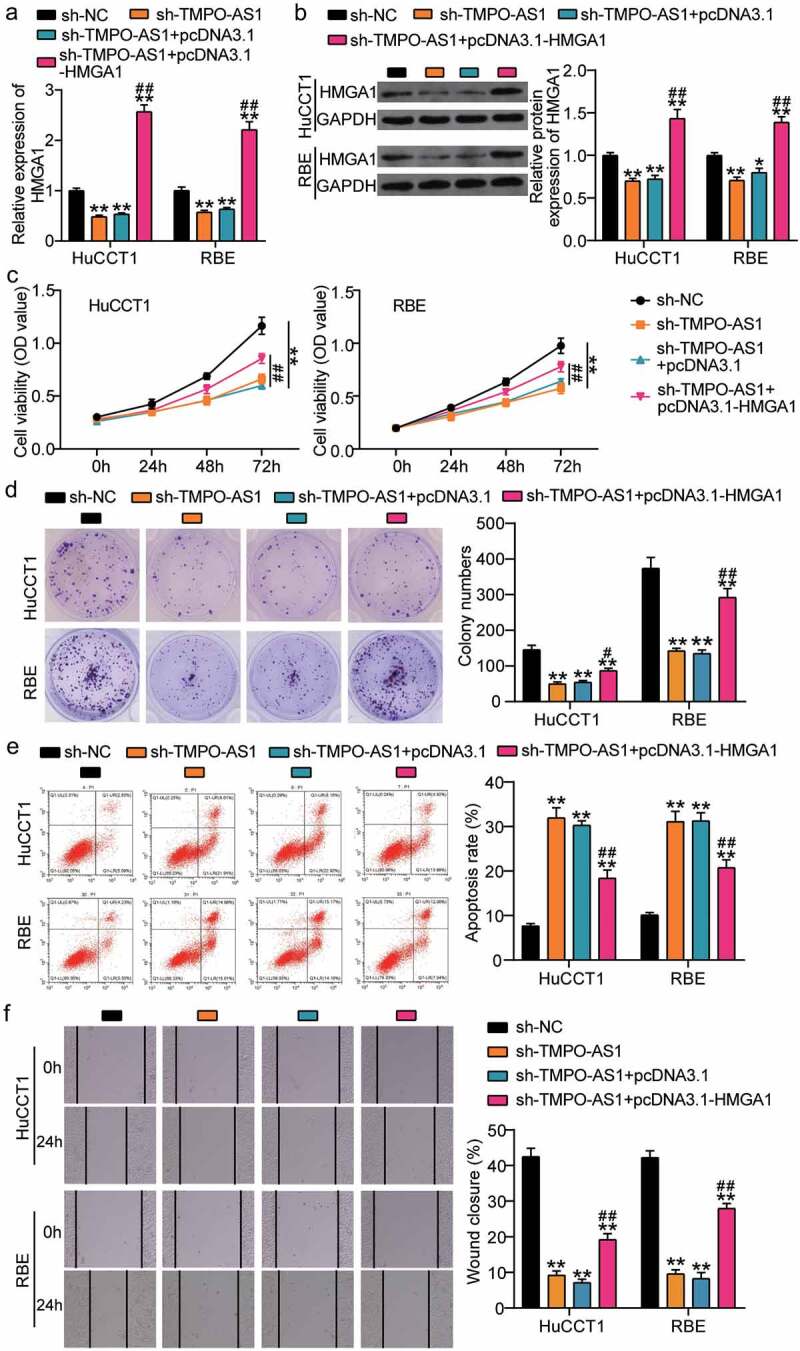
HMGA1 overexpression relieved the effect of sh-TMPO-AS1 on CHOL cells. (a-b) qRT-PCR (a) and Western blotting (b) identified the expression of HMGA1 in HuCCT1 and RBE cells. (c) CCK8 assay detected cell proliferation in HuCCT1 and RBE cells. (d) Colony formation assay assessed the colony formation capability of HuCCT1 and RBE cells. (e) Flow cytometry assay measured the cell apoptosis rate of HuCCT1 and RBE cells. (f) Wound healing assay identified the change of cell migration in HuCCT1 and RBE cells. sh-TMPO-AS1, siRNA targeting TMPO-AS1. NC, negative control. pcDNA3.1-HMGA1, HMGA1 overexpression vectors. **P < 0.01 vs. sh-NC. #P < 0.05, ##P < 0.01 vs. sh-TMPO-AS1.

To identify the mechanism of action of TMPO-AS1 in CHOL, we transfected sh-TMPO-AS1 and pcDNA3.1-HMGA1 overexpression vectors into CHOL cells. The results of qRT-PCR and Western blotting showed that sh-TMPO-AS1 induced a decrease in HMGA1 expression in HuCCT1 and RBE cells, however, pcDNA3.1-HMGA1 upregulated HMGA1 expression in HuCCT1 and RBE cells (Figure 5(a,b)). Furthermore, the CCK8 assay showed that pcDNA3.1-HMGA1 relieved the decrease in cell proliferation caused by sh-TMPO-AS1 knockdown in HuCCT1 and RBE cells (Figure 5(c)). Similar to the CCK8 results, the inhibitory effect of sh-TMPO-AS1 on colony information was partly reversed by co-transfecting pcDNA3.1-HMGA1 (Figure 5(d)). For cell apoptosis, the increase in apoptosis rate caused by sh-TMPO-AS1 was reduced by pcDNA3.1-HMGA1 (Figure 5(e)). Finally, the wound healing assay revealed that pcDNA3.1-HMGA1 enhanced the ability of cell migration that was impaired by sh-TMPO-AS1 (Figure 5(f)). Overall, our results suggest that the inhibitory effect of TMPO-AS1 knockdown on the malignancy of CHOL cells was partly relieved by HMGA1 overexpression.

## Discussion

lncRNAs are key regulatory factors that participate in CHOL progression [9–11]. In this study, TMPO-AS1, a lncRNA, was shown to be upregulated in CHOL cells; contributing to cell proliferation, colony formation, and cell migration, while suppressing cell apoptosis. Additionally, we found that let-7 g-5p could bind to TMPO-AS1 and HMGA1; subsequently deducing that TMPO-AS1 could suppress let-7 g-5p expression to upregulate HMGA1. Moreover, the reduced malignancy of CHOL, resulting from TMPO-AS1 silencing, was relieved by HMGA1 overexpression.

The oncogenic function of TMPO-AS1 has been reported in multiple cancers. For example, cytoplasmic TMPO-AS1 absorbed miR-577 to upregulate RAB14, thereby promoting the proliferation and migration of cervical cancer cells [16]. Another study explored the effect of TMPO-AS1 on gastric cancer and showed that TMPO-AS1 facilitated cell proliferation, cell migration, and angiogenesis in gastric cancer cells by targeting the miR-126-5p/BRCC3 axis to regulate the PI3K/Akt/mTOR pathway [35]. Here, we revealed, for the first time, the function of TMPO-AS1 in CHOL. We showed that TMPO-AS1 knockdown suppresses cell proliferation, colony formation, and migration, but promotes cell apoptosis. Our findings identified the function of TMPO-AS1 in CHOL and promote it as a potential biomarker for this disease.

The competing endogenous RNA (ceRNA) mechanism, first proposed by Salmena et al. in 2011, revealed that lncRNA could compete for miRNA as a natural miRNA sponge to regulate the mRNA that was targeted by miRNA [36]. Many studies have confirmed this mechanism in cancer, including CHOL. For example, lncRNA TTN-AS1 was found to sponge miR-320a to upregulate neuropilin-1, which resulted in increased proliferation and migration in CHOL cells [9]. Zhang et al. [37] discovered the role of lncRNA LOXL1-AS1 as a ceRNA upregulated ATP-binding cassette transporter A1 through sponging miR-324-3p, thereby exhibiting oncogenic function in CHOL. Here, we used bioinformatics analysis to predict the interaction between TMPO-AS1, let-7 g-5p, and HMGA1 in CHOL. Cell function experiments showed that let-7 g-5p acted as a bridge to connect TMPO-AS1 and HMGA1 by the ceRNA mechanism, showing that TMPO-AS1 upregulated HMGA1 levels in CHOL cells by sponging let-7 g-5p. Moreover, the inhibitory effect of TMPO-AS1 knockdown on CHOL malignancy was partly relieved by the upregulation of HMGA1.

HMGA1 was first discovered in cervical cancer cells in 1983 by Lund et al. [24]. Subsequent studies found that HMGA1 expression was elevated to promote the progression of malignant cancers, including breast cancer [38], colon cancer [39], and human uterine serous carcinomas [40]. In cells from CHOL patients, HMGA1 was found to be overexpressed, which promoted colony formation and resistance to gemcitabine treatment [28]. Recently, Song et al. also found the promoting function of HMGA1 in CCA cell proliferation, invasion, and xenograft tumor growth [29]. Previous studies have shown that HMGA1 is an oncogene in CHOL and, in our study, we found HMGA1 to be downstream of TMPO-AS1; hence, we used the HMGA1 overexpression vectors to reduce the effect of TMPO-AS1 knockdown on CHOL cells. Our results show that HMGA1 overexpression partially reduces the inhibitory effect of TMPO-AS1 knockdown on CHOL cells, which enriches current knowledge on the regulatory mechanism of HMGA1 in CHOL.

Our study also has many limitations for the application of the TMPO-AS1/let-7 g-5p/ HMGA1 axis in CHOL diagnosis and treatment. The number of clinical samples in this study was only 36; therefore, more samples are needed to reveal the potential correlation between the TMPO-AS1/let-7 g-5p/HMGA1 axis and clinical characteristics of CHOL such as prognosis. In addition, the lack of in vivo experiments in this study limits the clinical application of the TMPO-AS1/let-7 g-5p/ HMGA1 axis. Consequently, a thorough exploration of the effect of the TMPO-AS1/let-7 g-5p/HMGA1 axis in an in vivo animal model should be pursued for future studies.

## Conclusion

In this study, we discovered a positive effect of TMPO-AS1 on CHOL cells by improving cell proliferation, colony formation, and cell migration, but impairing cell apoptosis. In addition, we proved that TMPO-AS1 could sponge let-7 g-5p to upregulate HMGA1 to participate in the malignancy of CHOL cells. Our findings suggest a novel approach for CHOL diagnosis and therapy.

## Data Availability

All data are included in this published article.

## Highlights

- lncRNA TMPO-AS1 was an oncogenic lncRNA in CHOL.
- lncRNA TMPO-AS1 could sponge let-7g-5p to regulate HMGA1 in CHOL cells.
- HMGA1 overexpression partly relieved the effect of lncRNA TMPO-AS1 knockdown on CHOL cells.
